# High Throughput Genomic Screen Identifies Multiple Factors That Promote Cooperative Wnt Signaling

**DOI:** 10.1371/journal.pone.0055782

**Published:** 2013-01-31

**Authors:** Mayumi F. Miller, Ethan David Cohen, Julie E. Baggs, John B. Hogenesch, Edward E. Morrisey

**Affiliations:** 1 Department of Cell and Developmental Biology, University of Pennsylvania, Philadelphia, Pennsylvania, United States of America; 2 Department of Medicine, University of Pennsylvania, Philadelphia, Pennsylvania, United States of America; 3 Institute for Regenerative Medicine, University of Pennsylvania, Philadelphia, Pennsylvania, United States of America; 4 Cardiovascular Institute, University of Pennsylvania, Philadelphia, Pennsylvania, United States of America; 5 Department of Pharmacology, University of Pennsylvania, Philadelphia, Pennsylvania, United States of America; 6 Departmentof Medicine, Division of Endocrinology and Metabolism, University of Rochester School of Medicine and Dentistry, Rochester, New York, United States of America; Comprehensive Pneumology Center, Germany

## Abstract

Previous studies have demonstrated that certain Wnt ligands can promote high levels of cooperative signaling in a cell type specific manner. To explore the underlying mechanism of this cooperative Wnt signaling, we performed a high-throughput screen of more than 14,000 cDNAs to identify genes that promote cooperative Wnt signaling in the context of a single Wnt ligand, Wnt2. This screen identified several homeobox factors including *Msx2*, *Nkx5.2*, and *Esx1*, in addition to other factors known to promote Wnt signaling including *Pias4*. Generation of dominant-active or dominant-negative forms of Msx2 indicate that the mechanism by which homeobox factors cooperatively promote Wnt signaling is through their ability to repress gene transcription. These data identify a broad homeobox code, which acts to increase Wnt signaling through transcriptional repression.

## Introduction

Several studies have demonstrated that specific combinations of Wnt ligands cooperate to allow for spatial and temporal specificity of Wnt activity during development [1], [2]. Some of these ligands such as Wnt2 and Wnt7b are expressed in complementary patterns during organ development. In the developing mouse lung, *Wnt2* is expressed in the mesenchymal compartment, while *Wnt7b* is expressed in the epithelial compartment and these two ligands promote very high levels of signaling in lung mesenchyme [3]. This cooperative signaling by Wnt2 and Wnt7b is necessary and sufficient to promote smooth muscle and distal endoderm progenitor cell development in the lung. Moreover, this cooperative activation of Wnt signaling, while requiring beta-catenin expression, is not the result of increased beta-catenin stabilization. Precedent for increased Wnt signaling without a concomitant increase in beta-catenin protein levels has been demonstrated by the activity of beta-catenin co-activators, including p300 and pontin52, both of which cause a significant increase in Wnt reporter activation without increasing beta-catenin stabilization [4], [5].

Homeobox transcription factors are a large group of transcriptional regulators that are critical for embryonic development. Homeobox genes in the same family are all related by a highly homologous homeobox DNA binding domain and these factors can function as transcriptional activators or repressors. Several homeobox genes have been implicated in enhancing Wnt signaling. For example, Pitx2, a paired-homeodomain containing transcription factor (Prd), can directly bind to beta-catenin and synergistically activate the promoter of *Lef-1* [6]. The homeobox factor Hhex binds to a Wnt signaling repressor, Sox13, displacing it from Tcf3/4 to relieve transcriptional repression and allow for Wnt mediated transcriptional activation [7]. Such studies indicate the importance of transcription factor interactions in promoting Wnt signaling activity.

Given that our previous studies showed a specific cooperative interaction between Wnt2 and Wnt7 families of ligands that promote high levels of activity in the developing lung mesenchyme, we performed a high throughput cDNA overexpression screen to interrogate the mechanism of this cooperation [3]. Previous studies have demonstrated the ability of these screens to identify key regulatory factors in signaling pathways [8]. From this screen, we identified multiple homeobox factors that promote high levels of signaling in the presence of Wnt2. Such studies indicate a broad homeobox “code” that may be important for Wnt signaling activity in development and stem cell biology.

## Results

**Figure 1:**
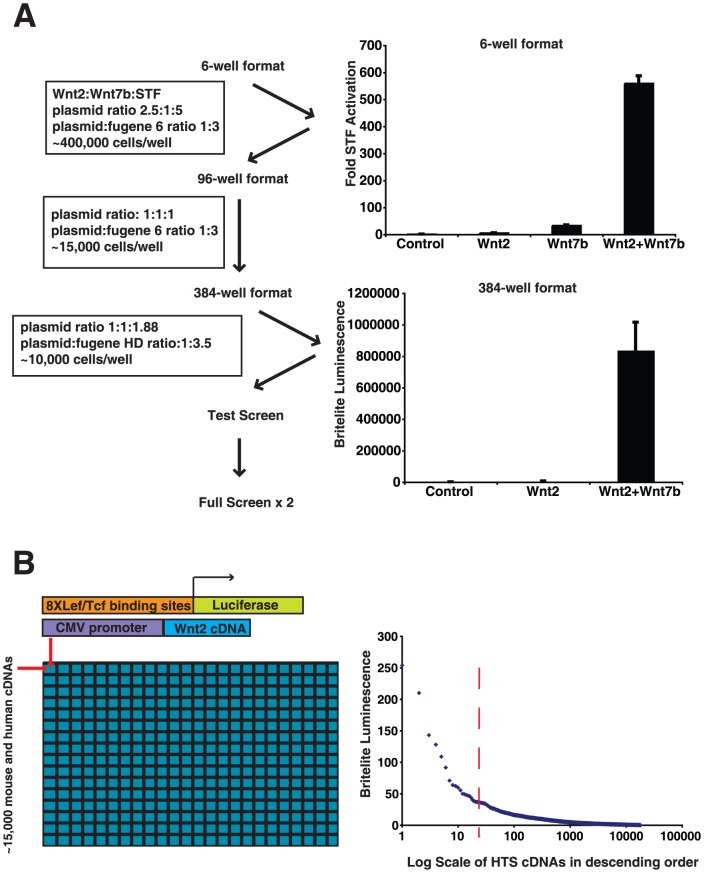
High throughput genomic screen for identification of factors cooperating with Wnt2 signaling activity. (A) The standard 6-well assay was first optimized for 96-well format and then for the high throughput 384-well format. (B) Diagram of how the 384-well HTS screen was performed. Our cutoff was at 35-fold higher than the median of each plate. Each 384-well plate contained internal positive and negative controls.

Previous work from our lab has demonstrated the importance of the cooperation between *Wnt2* and *Wnt7b* in the development of the early mouse lung [3]. To determine factors that could act upstream or downstream of *Wnt7b*, we performed a high throughput screen (HTS), utilizing the Mammalian Genome Collection (Open Biosystems), which includes 9,017 mouse and 5,445 human full length cDNAs driven by the CMV promoter arrayed in 46×384-well plates. We performed the screen in RFL6 cells, a rat lung fibroblast cell line used in our previous work demonstrating Wnt2-Wnt7b cooperative signaling [3]. To perform this screen, we first scaled down our SuperTOPFLASH (STF) Wnt cooperation assay to a 384-well plate format, from a 6-well plate format [9]. To do this, we scaled down into a 96-well plate format, and then scaled down to a 384-well plate format, optimizing plasmid and transfection reagent ratios as well as cell number (Fig. 1A). Additional steps were taken to ensure that the signal to noise ratio between control transfected cells and cells transfected with Wnt2+Wnt7b plasmids was significant enough that the screen produced consistent and reproducible results.

To perform the screen, a transfection mix containing control expression plasmids as well as the STF reporter, along with the transfection reagent Fugene HD, was added to 384-well plates containing the Mammalian Genome Collection in expression vectors (Fig. 1B). RFL6 cells were then added to the wells and after a 48-hour incubation period, the STF activity was quantified utilizing the BriteLite assay and the Perkin Elmer Envision luminometer. The screen was performed twice and the STF measurements were normalized and averaged.

**Figure 2:**
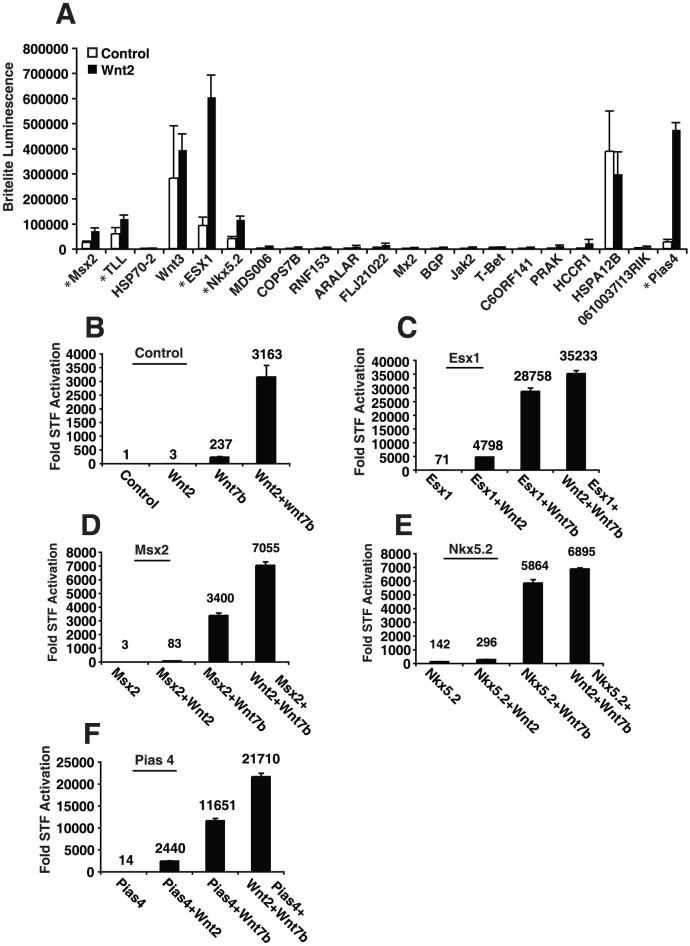
Homeobox factors Msx2, Esx1, Nkx5.2 as well as Pias4 were identified in a HTS screen for factors that cooperate with Wnt2. (A) The 23 hits that were obtained in the HTS screen were repeated in a 96-well format. Two of the hits, Wnt3 and HSPA12B, activated the STF reporter in the absence of Wnt2 and did not cooperatively promote signaling in the presence of Wnt2. The five genes specifically acting with Wnt2 are denoted by (*). Wnt2 plus Wnt7b cooperatively activates STF (B). Expression of Esx1 (C), Msx2 (D), Nkx5.2 (E), and Pias4 (F) further promotes this cooperative signaling in the presence of Wnt2 or Wnt7b. Data represent average of three assays performed in triplicate + S.E.M.

A cut-off point of 35-fold or higher than the median value of each 384-well plate was chosen to identify cDNAs from the screen for further investigation. This resulted in 23 unique genes to characterize (Fig. 1B and Figure S1). From the 23 genes, we tested whether the cDNAs could activate the STF reporter by themselves or if they would activate the SuperFOPFLASH (SFF) reporter, which has mutated Lef/Tcf sites and thus should not be activated by Wnt signaling activity. Of the 23 cDNAs, five specifically activated the STF reporter in combination with Wnt2 (Fig. 2A). Of these five, three encoded homeobox containing genes (*Esx1*, *Msx2*, and *Nkx5.2*) and one encoded the sumoylation factor *Pias4* which is known to regulate and alter Wnt/beta-catenin signaling [10], [11], [12], [13], [14]. All of these factors cooperatively enhanced Wnt signaling when co-expressed with either *Wnt2* or *Wnt7b* (Fig. 2B, C, D, E, F).

**Figure 3:**
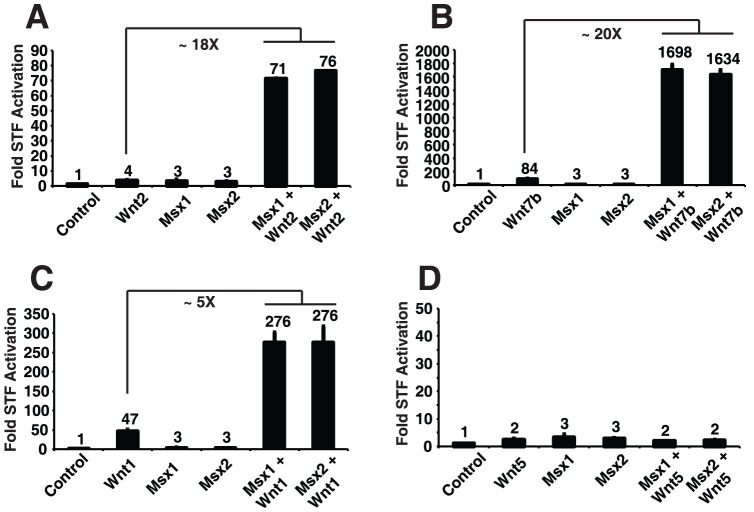
Promotion of cooperative Wnt2-Wnt7b signaling by homeobox transcription factors is Wnt ligand specific. Wnt cooperative signaling assays were performed in RFL6 cells in the presence or absence of Wnt2 (A), Wnt7b (B), Wnt1 (C), or Wnt5a (D). Data represent average of three assays performed in triplicate + S.E.M.

Of the three homeobox genes, we chose to focus on *Msx2*, and its highly related family member *Msx1*, since previous reports have demonstrated that these factors regulate Wnt signaling, particularly in mesenchymal cells [15], [16], [17], [18], [19]. Additional support for a role for Msx genes in the cooperation between Wnt2 and Wnt7b is that both *Msx1* and *Msx2* are expressed in the developing mouse lung from E10.5-E15.5 in the lung mesenchyme (Figure S2). To determine whether the *Msx1/2* genes enhance the activity of other Wnt ligands, we expressed *Msx1/2* along with *Wnt2, Wnt7b*, *Wnt1,* or *Wnt5a* in RFL6 cells, as outlined in Table 1. When co-expressed with Wnt2 and Wnt7b, the fold activation Msx1/2 were able to confer over the Wnt alone was 18 and 20 fold, respectively (Fig. 3A and B). However, with Wnt1 and Wnt5a, Msx2 only enhanced the signal by 5-fold for Wnt1, and had no positive effect on Wnt5a signaling (Fig. 3C and D). These data suggest that Msx1/2 enhance canonical Wnt signaling and that their affects are Wnt ligand specific.

**Table 1:** Outline of transfection protocol for Wnt2-Wnt7b and Msx factors.

	CMV beta-gal	Wnt2	Wnt7b	Msx	STF	Renilla
Control	0.417				0.208	0.104
Wnt2	0.313	0.104			0.208	0.104
Wnt7b	0.375		0.042		0.208	0.104
Wnt2+Wnt7b	0.271	0.104	0.042		0.208	0.104
Msx	0.344			0.073	0.208	0.104
Msx+Wnt2	0.24	0.104		0.073	0.208	0.104
Msx+Wnt7b	0.302		0.042	0.073	0.208	0.104
Msx+Wnt2+Wnt7b	0.198	0.104	0.042	0.073	0.208	0.104

We also expressed Msx2 with Wnt2 and Wnt7b in additional cells lines, including 3T3, 10T1/2, and HEK293 to determine if the cooperation could occur in additional mesenchymal, or epithelial, cell lines. Msx2 cooperates with Wnt2 and Wnt7b in both of the mesenchymal cell lines, 3T3 and 10T1/2, but not the epithelial cell line HEK293 (Figure S3). This is in agreement with previous data from out lab showing that the cooperation between Wnt2 and Wnt7b occurs specifically in mesenchymal cell types [3].

**Figure 4:**
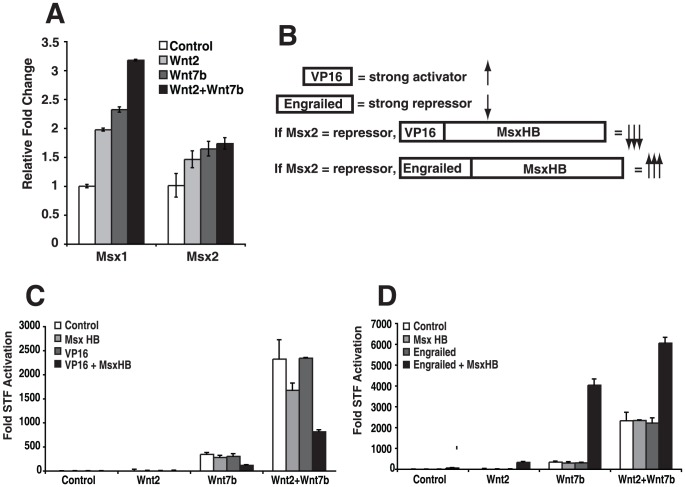
Msx1/2 expression is induced by Wnt2-Wnt7b and Msx represses gene transcription to promote Wnt2-Wnt7b cooperative signaling. Wnt2 and Wnt7b increase Msx1/2 gene expression in RFL6 cells (A). Diagram of Msx1 HB fusion proteins made with the VP16 activation domain and the engrailed repression domain (B). Expression of the MsxHB-VP16 fusion represses Wnt2-Wnt7b cooperative signaling (C), while expression of the MsxHB-engrailed fusion enhances Wnt2-Wnt7b cooperative signaling (D).

To determine whether Wnt2 or Wnt7b activity can affect the transcription of *Msx1* or *Msx2*, we performed qualitative RT-PCR (Q-PCR) on RFL6 cells expressing Wnt2 and/or Wnt7b. These studies show that expression of *Msx1* and *Msx2* are induced by ectopic Wnt2 and Wnt7b expression (Fig. 4A). Additionally, expression of *Msx1* and *Msx2* is decreased in Wnt2-/-;Wnt7b-/- mutant embryos at E10.5 (Figure S2b). Next, we wanted to determine how Msx1/2 affect the cooperation between Wnt2 and Wnt7b. To determine the activity of Msx1/2 in the context of Wnt2 and Wnt7b, we produced fusion constructs containing the *Msx1* homeobox domain (MsxHB) to construct MsxHB-Engrailed and MsxHB-VP16 expression plasmids to repress and activate Msx target genes, respectively. The MsxHB-VP16 construct, which we initially hypothesized would increase the cooperation between Wnt2 and Wnt7b, decreased the Wnt2-Wnt7b cooperative Wnt signaling. In contrast, the MsxHB-Engrailed construct increased Wnt2-Wnt7b cooperative Wnt signaling (Fig. 4B, C, D). Thus, our data suggests that Msx1/2, and possibly other homeobox factors, promote cooperative Wnt2-Wnt7b signaling through transcriptional repression of potential inhibitors of Wnt signaling.

## Discussion

Wnt signaling is a critical regulator of early development as well as stem cell differentiation and self-renewal. How the Wnt pathway signaling activity is regulated by interactions with other transcriptional modulators is still poorly understood. By performing a HTS screen using the majority of the coding genes in the mammalian genome, we have shown that several homeobox factors that cooperatively with certain Wnt ligands, including Wnt2 and Wnt7b, to promote high levels of Wnt signaling activity in mesenchymal cells. These homeobox factors encompass several subfamilies including the Msx and Nkx subfamilies. Our data also suggest that this cooperative increase in Wnt signaling activity is driven by the ability of homeobox factors to repress gene transcription, possibly through repression of inhibitors of Wnt signaling activity. Thus, homeobox factors may both promote and fine tune Wnt signaling in specific developmental contexts through inhibition of negative inputs into the Wnt pathway.

Wnt signaling has been described as a network rather than a single linear pathway [20]. Our data showing a broad role for homeobox factors in promoting Wnt signaling correlates with such a network assessment for Wnt signaling. Several previous reports have demonstrated roles for homeobox factors in the Wnt pathway. Prop1, which contains a Prd-like homeodomain, works in conjunction with Wnt signaling by interacting with beta-catenin to both activate and repress transcription in a tissue specific manner to allow for proper cell differentiation in the pituitary gland [21]. Alx4, another Prd-domain containing protein, binds to and physically interacts with Lef-1 in vitro, and has been shown to genetically interact with *Lef-1* in heart, head, and dorsal vessel development in vivo [22], [23]. Despite these examples, much is still not understood about how homeobox factors regulate Wnt signaling output. Our studies suggest that transcriptional repression may underlie at least part of the mechanism. Such repression could be through a simple inhibition of repressors of Wnt signaling allowing for maximal signaling output in certain contexts. Alternatively, homeobox factors may inhibit portions of the Wnt pathway that provide important negative feedback on signaling. Future studies will be needed to further assess how the homeobox factor family promotes Wnt signaling through transcriptional repression.

The HTS screen performed in these studies was done in the context of a specific ligand, Wnt2. However, most of the positive hits we obtained also showed significant cooperative increases with Wnt7b but not other ligands including Wnt1 or Wnt5a. These results further indicate that the large number of Wnt ligands evolved to perform specific functions in specific contexts. This is an important concept given the wide use of some recombinant Wnt proteins i.e. Wnt3a to demonstrate active Wnt signaling in multiple different contexts [24], [25], [26], [27]. Thus, our data suggest that the use of a Wnt ligand to study a given biological system in vitro should match with the expression pattern of the ligands used in vivo where possible.

## Materials and Methods

### High throughput genomic screen of the Mammalian Gene Collection

50ng of each cDNA from the Open Biosystems Mammalian Gene Collection was arrayed on 46×384-well plates. The final plasmid amount in each well was 155ng (50 ng MGC cDNA + 40 ng *Wnt2* expression plasmid + 75 ng SuperTOPFLASH). FugeneHD (Roche) was dispensed at a DNA:Fugene HD ratio of 1 µg:3.5 µL. 10,000 RFL6 cells were dispensed to each well. The transfected cells were grown in DMEM (Gibco), 10%FBS (Gibco), and 1%Antibiotic/Antimycotic (Gibco). Transfection master mixes containing plasmid/DMEM/FugeneHD were dispensed to each well using the Matrix Wellmate. 20 µl of RFL6 cells at 5×10^5^ cells/mL were then dispensed to each well using the Matrix Wellmate. For transfection controls, we utilized both a *Wnt7b* expression plasmid and a constitutively active *beta-catenin* mutant cDNA plasmid. Plates were incubated at 37°C and 5% CO_2_ for 48 hours. 35 µl of BriteLite (PerkinElmer) was dispensed to each well using a multi-channel pipette and allowed to incubate for 5 minutes. Luciferase values were then recorded using the PerkinElmer Envision plate reader. The screen was repeated, and the luciferase values for each well averaged. The median value of each plate was used to normalize the values between the 46 plates.

### VP16 and engrailed fusions with the Msx homeodomain

The VP16 activation domain (amino acids 411–456) was cloned into pcDNA3.1 using NheI and KpnI. The Xenopus engrailed repression domain (amino acids 1–297) was cloned into pcDNA3.1 using NheI and KpnI. MsxHB (amino acids 157–233 of mouse Msx1) was cloned into pcDNA3.1 and downstream of the activation and repression domains of the VP16 and Engrailed pcDNA3.1 constructs using KpnI and XhoI.

### Cell Transfections

RFL6, 10T1/2, 3T3, and HEK203 cells were plated into 24-well plates at a concentration of 1×10^5^ cells/mL. One day later, cells were transfected in triplicate using the below microgram amounts (calculated for 3.5 wells of a 24-well plate) and Fugene 6 at a ratio of 3 microliters Fugene 6∶1 micrograms plasmid. A pCMV-βGal plasmid was used as a control plasmid and to equalize total plasmid amounts per transfection. 48 hours following transfection, we performed a Dual-Luciferase Assay (Promega) to determine activation of the STF reporter and Renilla transfection efficiency. RFL6, 3T3, 10T1/2, and HEK293 cell lines were purchased from ATCC.

### Q-PCR

Cells and lung buds were lysed and RNA collected following the Trizol Reagent (Invitrogen) protocol. Following DNase treatment (Roche), cDNA was synthesized using the First-Strand cDNA synthesis kit (Invitrogen). QPCR was then performed using the below primers and SYBR green (Applied Biosystems).

Msx1: Forward 5’ TCCTCCTGGCCATCGCATCTTAAA, Reverse 5’ ATATTGGGAAGAGGTGGACAGGCA

Msx2: Forward 5’ ATTGAAGCCATGTGTTGGGCTTGG, Reverse 5’ ATATTGGGAAGAGGTGGACAGGCA

In situ hybridizations were performed as previously described [28].

## Supporting Information

Figure S1 **Most of the genes identified in the genomic screen cooperate with Wnt2 signaling.** Msx2, TLL, Pias4, ESX1, and Nkx5.2 cooperated with Wnt2 (grey bars) much more strongly than they were able to activate STF alone (white bars) or SFF alone (black bars) (A). Wnt3 and HSPA12B activated STF very strongly (white bars) and the addition of Wnt2 (grey bar) did not result in a cooperative increase of STF (B). Neither Wnt3 nor HSPA12B strongly activated SFF (black bars) (B). The other 16 genes identified in the genomic screen did show low levels of cooperation with Wnt2 (grey bars) but not to the same extent as the genes shown in A (C).
(EPS)

Figure S2 **Msx1 and Msx2 are expressed in the developing lung mesenchyme.** Q-PCR for Msx1 and Msx2 on isolated lung buds from E11.5 to E15.5 show robust expression of Msx1 and Msx2 during early lung development (A and B). In situ hybridization for Msx1 and Msx2 in wild type lung sections at E10.5 show Msx1 and Msx2 expression in the lung mesenchyme adjacent to the trachea (yellow dashed line) (C and D). Expression of Msx1 and Msx2 are decreased In Wnt2-/-;Wnt7b-/- mutants lungs at E10.5 (yellow dashed line) (E and F). E = esophagus, T = Trachea
(EPS)

Figure S3 **The ability of Msx2 to cooperate with Wnt signaling is specific to mesenchymal cells.** Msx2 is able to promote cooperative Wnt signaling through activation of the STF reporter in two additional mesenchymal cell lines, 3T3 and 10T1/2 (A and B), but not in the epithelial cell line HEK293 (C).
(EPS)
